# Vascularization and Bone Regeneration with 3D-Printed Composite Scaffolds in Rodent Critical-Size Calvarial Defects: Systematic Review

**DOI:** 10.3390/jfb17030115

**Published:** 2026-02-27

**Authors:** Milda Vitosyte, Melanie Tesing, Sarlota Galinauskaite, Vygandas Rutkunas, Ieva Gendviliene

**Affiliations:** 1 Institute of Odontology, Faculty of Medicine, Vilnius University, Zalgiris Street 117, LT-08217 Vilnius, Lithuania; melanie.tesing@gmail.com (M.T.); sarlota.galinauskaite@mf.stud.vu.lt (S.G.); vygandas.rutkunas@mf.vu.lt (V.R.); ieva.gendviliene@mf.vu.lt (I.G.); 2 Centre of Oral Bioengineering, Queen Mary University of London, London E1 4NS, UK; 3 Department of Restorative Dentistry, Barts Health NHS Trust, The Royal Dental Hospital, London E1 1FR, UK

**Keywords:** 3D printing, composite scaffold, bone regeneration, angiogenesis, vascularization, micro-CT, Microfil perfusion, critical-size calvarial defect, tissue engineering

## Abstract

Rapid vascularization is essential for bone regeneration in oral and maxillofacial surgery. This systematic review synthesised in vivo evidence on 3D-printed composite scaffolds in rodent critical-size calvarial defects quantified by Microfil perfusion and micro-CT. “Composite” was defined as an organic–inorganic construct within the printed scaffold (not a single-phase scaffold with a surface coating). PubMed, MEDLINE, and Web of Science Core Collection were searched for studies published from January 2014 to December 2025. Eligible studies compared composite scaffolds with non-composite (single-phase) scaffolds and/or empty controls and reported vascular outcomes (vessel number, vascularized area) together with bone outcomes (new bone area, bone volume fraction [BV/TV], and bone mineral density). Ten studies met the inclusion criteria. In outcome-specific exploratory analyses, composite scaffolds were associated with higher new bone area than comparators (*p* = 0.031). Functional modifications were associated with higher vascularized area (*p* = 0.025) and higher new bone area (*p* = 0.038), while dual-factor modifications showed the largest gain in new bone area (*p* = 0.002). Pore sizes ≥ 400 μm were associated with higher BV/TV (*p* = 0.029). Heterogeneity in designs, follow-up, and reporting, together with small sample sizes, precluded meta-analysis. Composite scaffolds appear promising, but standardised methodologies and improved reporting are needed to define optimal design features and support translation.

## 1. Introduction

Regenerative medicine increasingly relies on bone graft procedures, which have grown in popularity. Globally, over four million bone graft or bone substitute surgeries are performed annually [1]. As dental implants have become more popular, bony reconstructions have increased in oral and maxillofacial surgery. With a compound annual growth rate of 7.7% expected to reach USD 1.8 billion by 2029 [2], the global dental bone substitute market was valued USD 450 million in 2020.

Clinically, predictable vascularized bone formation is critical for alveolar ridge augmentation, sinus floor elevation, peri-implant defects, and reconstruction of craniofacial defects. Although bone naturally repairs itself, critical-sized defects usually call for the use of bone grafts and substitutes [3,4]. Dental bone defects may result from trauma, periodontal disease, tooth extractions due to caries, apical periodontitis, or other pathologies. Autografts consistently enhance bone volume and quality, which are essential for the placement and stability of implants [5,6], yet limited availability, donor-site morbidity, and morphological restrictions constrain their clinical relevance [5]. These difficulties have spurred new approaches for bone regeneration and developments in bone tissue engineering [7].

Bone tissue engineering aims to develop scaffolds that serve as structural frameworks to support bone growth while providing mechanical stability. Composite scaffolds, which blend organic and inorganic components, show promise by emulating the architecture and properties of natural bone [8]. In this review, a 3D-printed composite scaffold denotes an additively manufactured scaffold comprising both an organic (polymeric) phase and an inorganic phase within the construct [9]. Functional modification refers to the incorporation of additional angiogenic and or osteogenic cues applied to the scaffold beyond base composition and architecture (for example, growth factors, cells, ions, small molecules, or surface-applied coatings). A dual-factor strategy denotes the use of two distinct modifications within the same scaffold design, in contrast to single-factor modification [10]. Their internal structure can be precisely tailored, and their biological, chemical, and physical characteristics can be enhanced through the incorporation of bone-specific growth factors and stem cells to optimise tissue regeneration [11,12]. However, key aspects of scaffold design remain debated across the field, including how strongly pore size and architecture drive vascular ingrowth versus osteogenesis, and whether functional modifications consistently translate into superior outcomes across different materials and study protocols [11,12].

Advancements in imaging have further accelerated bone regeneration research. The integration of micro-CT with “Microfil” perfusion enables high-resolution 3D assessment of bone quality, scaffold integration, and vascularity [13]. “Microfil” is a radiopaque contrast agent used to visualise blood vessel architecture, and multimodal imaging supports precise localisation and quantification of vascularised regions [14]. At the same time, differences in imaging settings, segmentation, and reporting can complicate comparisons between studies, even when similar endpoints are targeted [13,14]. Quantitative readouts including vessel number (VN), vascularised area (VA), bone volume fraction (BV/TV), bone mineral density (BMD), and new bone area (NBA) facilitate structured comparisons across studies [13].

To maximise comparability and reduce methodological heterogeneity, we predefined a narrow scope restricted to rodent critical-size calvarial defects assessed using Microfil perfusion with micro-CT. The calvarial model is widely used and ethically efficient for craniofacial research, offers standardised defect geometry, is largely non-load-bearing, and typically does not require fixation hardware, enabling more direct comparisons of scaffold architecture and composite design under relatively consistent mechanical conditions [15]. In contrast, long-bone models are strongly influenced by load-bearing biomechanics, fixation strategy, and marrow-associated healing compartments, which can confound scaffold-specific effects and substantially increase between-study heterogeneity [16].

This systematic review therefore investigates, using “Microfil” perfusion and micro-CT, the impact of 3D-printed composite scaffolds on new bone formation and vascularisation in rodent critical-sized calvarial defects, addressing a notable void in the current literature. Overall, the included evidence indicates that 3D-printed composite scaffolds commonly support improved bone formation, while vascular outcomes are more variable. Larger pore sizes showed the most consistent association with BV/TV, whereas VA demonstrated only a non-significant positive trend, and dual-factor (two-modification) strategies showed the most consistent signals of benefit [8,11,12,13,14]. Based on mechanistic and preclinical evidence, we predefined the following hypotheses: (1) larger pore sizes (≥400 μm) are associated with improved vascularization and bone formation compared with smaller pores; (2) 3D-printed composite scaffolds achieve superior vascular and bone outcomes compared with non-composite (single-phase) scaffolds and/or controls; and (3) dual-factor functionalization yields superior outcomes compared with single-factor functionalization. These comparisons were specified a priori to structure data extraction and synthesis.

## 2. Materials and Methods

### 2.1. Eligibility Criteria

Predefined inclusion and exclusion criteria were applied to ensure a consistent study set representative of vascularisation and bone-regeneration effects in 3D-printed composite scaffolds. Eligibility and the research question were defined a priori using the PICOS framework: rodents with critical-size calvarial defects (P); 3D-printed composite scaffolds, with or without angiogenic/osteogenic factors (I); non-composite (single-phase) scaffolds and/or unmodified 3D-printed composite scaffolds (C); VN, VA, BV/TV, BMD, and NBA quantified by ‘Microfil’ perfusion and micro-CT (O); and in vivo animal studies with mandatory ‘Microfil’ perfusion and micro-CT analysis, with or without histology (S). This framework was predefined to maintain a craniofacial translational focus and to ensure that vascular and bone outcomes were quantified using comparable three-dimensional imaging endpoints across studies.

A 3D-printed composite scaffold was defined as an additively manufactured scaffold comprising both an organic (polymeric; natural or synthetic) phase and an inorganic phase within the construct. Non-composite (single-phase) scaffolds comprised one material phase only. Bioceramic-only scaffolds with surface coatings were not classified as composites unless an additional organic phase was incorporated into the scaffold construct; coatings were treated as functional modifications.

Studies were included if they were in vivo rodent calvarial defect models evaluating vascularisation in 3D-printed composite scaffolds composed of inorganic and organic synthetic materials using ‘Microfil’ perfusion and micro-CT. Studies were excluded if they were reviews, in vitro, non-rodent, lacked ‘Microfil’ perfusion and micro-CT, or used scaffold types not aligned with the predefined intervention criteria. The protocol was registered in PROSPERO (CRD42024562748); reporting followed PRISMA and the review protocol adheres to PRISMA guidelines [17].

### 2.2. Literature Search

**Figure 1:**
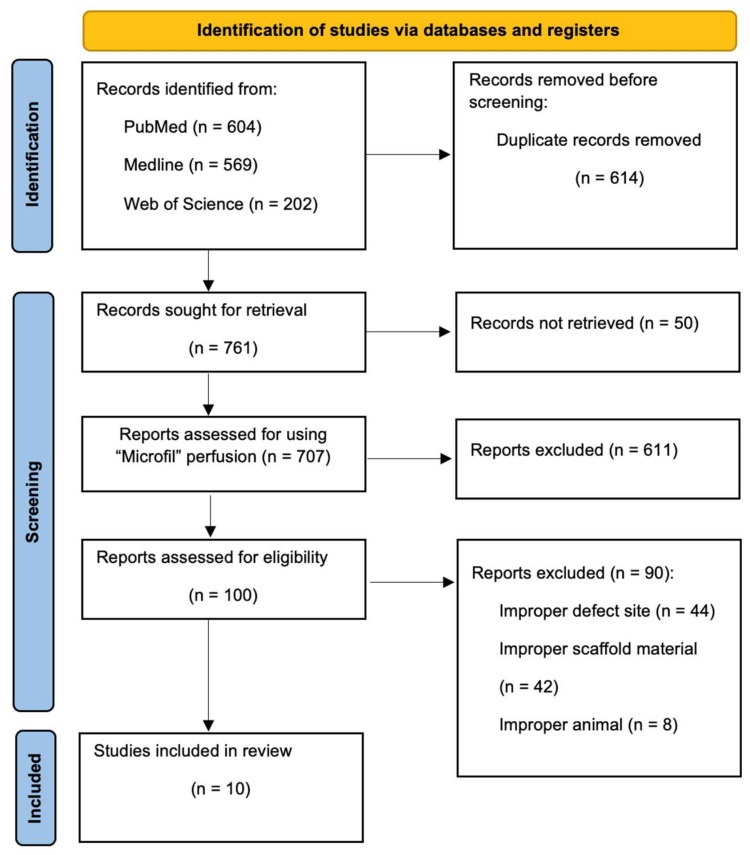
PRISMA flow diagram of the article selection procedure.

An electronic search was conducted in PubMed, Medline and Web of Science Core Collection databases from January 2014 to December 2025 (final search date 6 February 2026), limited to English language publications with available full texts. Search terms combined scaffold/composite and 3D-printing concepts with anatomical (calvarial/skull), perfusion (Microfil/vascular casting), and imaging (micro-CT/μCT) terminology. The following keywords were used: (calvar* OR cranial OR skull) AND (rodent* OR rat OR mouse OR murine) AND (scaffold* OR “3D print*” OR biomaterial*) AND (vasculari* OR angiogen*) AND ((Microfil OR Micro-Fil OR MV-122 OR “vascular cast*” OR “radiopaque polymer”) OR (“micro-CT” OR microCT OR μCT OR “micro computed tomograph*” OR “x-ray microtomography”)) NOT (review* OR “systematic review*” OR “in vitro” OR “ex vivo”). Figure 1 shows the flow chart diagram of the present study selection according to PRISMA guidelines [17]. Because perfusion media are poorly indexed, screening included full-text keyword searches for Microfil/Micro-Fil/MV-122/“vascular casting”. The PICOT question asked whether in rodents with critical-size calvarial defects, 3D-printed composite scaffolds improve vascularisation compared with alternative designs within 4 to 12 weeks.

### 2.3. Study Selection

Two reviewers independently screened titles and abstracts, and full texts were assessed against the eligibility criteria; a third expert resolved disagreements. As ‘Microfil’ perfusion was seldom noted in abstracts, potentially eligible records underwent full-text searches for “Microfil”, “Micro-Fil”, “MV-122”, and “vascular casting” before final inclusion. Systematic reviews, reviews, and in vitro studies were not considered.

### 2.4. Population, Type of Interventions and Outcome Selection

The population consisted of rodents in which critical-size calvarial defects were repaired using 3D-printed composite scaffolds and evaluated for vascularisation using ‘Microfil’ perfusion and micro-CT. Studies were analysed for vascularisation and bone regeneration outcomes (VN, VA, BV/TV, BMD, and NBA) in relation to scaffold material, additives, design and geometry, and timing of ‘Microfil’ perfusion.

### 2.5. Data Items

Extracted information included animal age/sex/strain (Sprague–Dawley/Wistar), sample size and groups, defect details, analysis methods, timing of ‘Microfil’, micro-CT and histology, outcomes (VN, VA, BV/TV, BMD, and NBA), and scaffold characteristics (dimensions, geometry, pore size, porosity/strength, and composition). Scaffolds were categorised by composition (composite vs. non-composite) and by modification strategy (unmodified vs. single-factor vs. dual-factor). Modifications were defined as growth factors, cells, ions, small molecules, or other additions applied to the scaffold (including surface coatings) and were classified as single-factor or dual-factor. To enable predefined subgroup comparisons, we extracted and coded pore size category (<400 μm vs. ≥400 μm), scaffold composition (composite vs. non-composite), and modification strategy (unmodified vs. single-factor vs. dual-factor).

For vascularisation, area-based vascularised area (VA, %) and vessel number (VN) were treated as the primary endpoints because these were most consistently reported across studies. When studies reported conceptually related but non-equivalent Microfil-derived vascular endpoints (for example, vascular volume fraction, VV/TV, vascular volume, and connectivity), these were extracted as secondary outcomes and synthesised descriptively, without conversion to VA/VN due to differences in region-of-interest definitions and segmentation approaches. For bone outcomes, quantitative comparisons were restricted to endpoints reported in directly comparable formats (for example, BV/TV in %, and BMD reported in calibrated density units); relative or non-calibrated BMD units were extracted but not pooled statistically.

### 2.6. Data Collection Process

Exploratory, predefined comparisons were performed across three design dimensions (pore size category, composite status, and modification strategy). Analyses were outcome-specific and restricted to studies reporting extractable quantitative data for the endpoint of interest. Data were extracted from included reports using a piloted, standardised extraction form. When numerical outcome values were not reported in text or tables, or when values were inconsistent between the text and figures, data were extracted from figures using plot digitisation and treated as approximate estimates.

### 2.7. Statistical Analysis

Data were analysed using S-Plus 8.0 for Linux (TIBCO Software Inc., Palo Alto, CA, USA). A two-sided *p* value ≤ 0.05 was considered statistically significant. Descriptive statistics were calculated for vascularisation and bone outcomes (VN, VA, BV/TV, BMD, NBA and porosity). Group differences in VN, VA, BV/TV, BMD and NBA by pore size, composite status, and presence or number of modifications were tested with independent samples *t* tests, applying Welch correction for VA when variances were unequal. Quantitative tests were performed only when endpoint definition and units were directly comparable across studies. Otherwise, outcomes (for example, VV/TV, connectivity, fold-change VA, or relative BMD) were summarised descriptively. Principal summary measures were the difference in means (reported with 95% confidence intervals) and standardised mean differences (Cohen’s d) for group comparisons. Due to the small number of studies and marked heterogeneity, a conventional meta-analysis was not performed.

### 2.8. Reporting Bias Assessment

SYRCLE’s risk of bias tool [18] was used to evaluate the risk of bias in each of the included animal studies. This approach is consistent with previous studies [19]. Since it was impossible to acknowledge each factor’s weight for the overall assessment, an overall risk of bias is not presented.

## 3. Results

A total of 1375 records were identified. After deduplication, 761 records were sought for retrieval, and 50 full texts were unavailable. Among the 707 retrieved reports, 611 were excluded because Microfil perfusion was not performed or not reported. The remaining 100 full texts were assessed against the predefined eligibility criteria, and 90 were excluded for reasons unrelated to Microfil (e.g., ineligible defect site, scaffold eligibility, or non-rodent models), leaving 10 included studies.

### 3.1. Study Characteristics

#### 3.1.1. Animals

All included studies employed rodent calvarial critical-size defect models (Table 1). Eight studies [20,21,22,23,24,25,26,27] employed male Sprague–Dawley rats (ages 8 to 13 weeks), and one study [28] used 4–5-month-old Wistar male rats. One study [29] used female Sprague–Dawley rats. With this single exception, all experiments used male rats; therefore, sex effects could not be assessed quantitatively. After excluding articles based on “improper defect site,” “improper scaffold material,” and lack of full-text availability, ten articles remained. All studies used male rats, with both Sprague–Dawley and Wistar strains represented; one study used female Sprague–Dawley rats.

#### 3.1.2. Sample Size, Study Groups, and Defect Features

Sample sizes ranged from 12 [23,26] to 126 [25]; one study [28] did not report a sample size. The number of study groups varied from 2 [26] to 6 [28]. Six studies included control groups [20,24,25,26,27,28]. Six studies created two defects per rat (5 mm diameter) [20,21,22,23,24,26], and four created a single defect per rat (5 mm [27,29], 6 mm [25] or 8 mm [28]).

#### 3.1.3. Analysis Methods

All included studies performed ‘Microfil’ perfusion, histological analysis and micro-CT. Additional analyses included fluorescence labelling [20,21,23,24,27] histomorphometry [23,28], immunohistochemistry [21,27,29], immunofluorescence [24], and biomechanical testing [25].

#### 3.1.4. Timing of ‘Microfil’ Perfusion, Micro-CT, and Histology

Studies performed ‘Microfil’ perfusion and micro-CT at various times: 4 weeks [28,29], 6 weeks [25,27], 8 weeks [20,21,23,24], 12 weeks [22,27], or 4 and 8 weeks [26]. One study performed micro-CT for bone regeneration at 4 and 12 weeks [25]. Additionally, one study performed micro-CT at 2 and 4 weeks [29].

#### 3.1.5. Vascularisation and New Bone Formation Analysis

All but one study [20] reported VA after scaffold implantation; four [21,22,25,26] also reported VN. One study [28] expressed VA in fold increase, and another [26] provided VA at two time points (4 and 8 weeks). Additional VA was reported at 4 weeks [29] and at 12 weeks [27]. Yang et al. [29] quantified vascularisation as vascular volume fraction (VV/TV, %) at 4 weeks, rather than area-based VA. For quantitative comparisons (Table 2), we pooled area-based VA reported as an absolute value (%) and VN, whereas VV/TV and other Microfil-derived vascular metrics (for example, connectivity) were synthesised descriptively because they are not directly interchangeable with VA/VN. Accordingly, this study contributed to the descriptive synthesis of vascular outcomes but was not included in VA-based quantitative comparisons. Most studies quantified new bone formation using different measures (Table 1), with NBA being the most frequently reported area-based endpoint and therefore carried forward for quantitative comparison when available. Quantitative comparisons were restricted to outcomes reported in directly comparable formats (BV/TV and NBA, and BMD only when reported in calibrated units). Relative or non-calibrated BMD was extracted but not pooled. Six studies [21,22,23,24,25,26] reported NBA, seven [20,22,24,25,26,27,29]—BV/TV, and three [21,25,26]—BMD. Some measured outcomes at multiple time points (2, 4, 8, or 12 weeks). Because fewer than three studies per strain reported each primary outcome, formal Sprague–Dawley versus Wistar subgroup analysis was not performed; strain was treated qualitatively as a potential source of heterogeneity.

### 3.2. Analysis of Included Studies

#### 3.2.1. Scaffold Design

The analysed studies presented diverse scaffold designs, ranging from 5 to 12 mm in length and 0.6–5 mm in height, except for one 5 mm membrane [26] with no reported height. Scaffold structures varied from disordered, irregular to uniform, well-defined with porosities between 58.8% [26] and 91% [23]. Pore sizes ranged from 20 μm [21] to 1000 μm [25], with one study incorporating both macropores and micropores [21]. Because outcome reporting was incomplete across studies (and some outcomes were not reported in a directly comparable quantitative format), the analyses in Table 2 are outcome-specific and based on subsets of the included studies, with the effective sample size varying by outcome. NBA was available in six studies and was therefore included in the NBA-specific rows of Table 2. Studies without NBA reporting were excluded from NBA comparisons. Statistical analysis (Table 2) showed that scaffolds with bigger pores (≥400 μm) had significantly higher BV/TV (t = −2.66, *p* = 0.029) and borderline higher VA (t = −2.28, *p* = 0.053) compared with small pores (<400 μm). BMD results were excluded due to missing small-pore data.

#### 3.2.2. Scaffold Composition

Composite status was not associated with differences in VN (*p* = 0.601), VA (*p* = 0.490), or BV/TV (*p* = 0.333). In contrast, NBA was higher in the composite group (t = 2.70, *p* = 0.031; 95% CI of the mean difference: 1.78 to 26.99). Given the small number of non-composite comparators and heterogeneous scaffold designs, this finding should be interpreted cautiously.

#### 3.2.3. Scaffold Modification

Statistically significant differences were observed for VA (t = 2.58, *p* = 0.025) and NBA (t = 2.23, *p* = 0.038) between modified and non-modified scaffolds; BV/TV showed a borderline difference (t = 2.03, *p* = 0.055) and VN a trend (t = 2.19, *p* = 0.051) (Table 2). Further analysis comparing single- and dual-factor modified scaffolds revealed statistically significant improvements in VN (*p* < 0.001), BV/TV (*p* = 0.046), and NBA (*p* = 0.002) with dual modifications. Dual-factor scaffolds demonstrated higher means for these parameters, supporting their enhanced efficacy in promoting vascularisation and bone regeneration.

### 3.3. Risk of Bias

**Figure 2:**
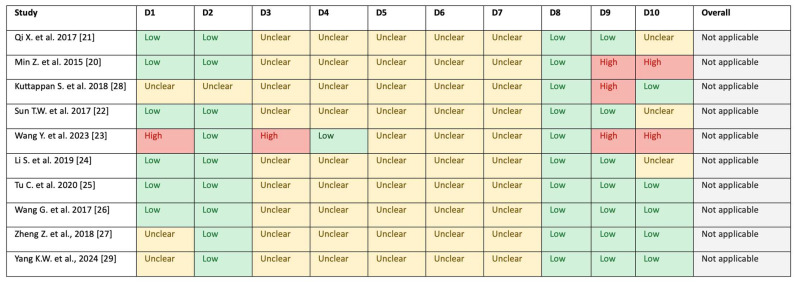
Risk of bias in the animal studies D1–D10 criteria. D1 random sequence generation; D2 baseline characteristics; D3 allocation concealment; D4 random housing; D5 blinding of caregivers and investigators; D6 random outcome assessment; D7 blinding of outcome assessors; D8 incomplete outcome data; D9 selective outcome reporting; D10 other sources of bias.

Three studies exhibited at least one high-risk item (Figure 2). [20] failed to quantify vascularisation and bone formation outcomes (VN, VA, BMD, BV/TV, and NBA) and reported ‘Microfil’ data for controls without specifying group sizes. [28] did not report VN, BV/TV or BMD and presented VA as a fold increase without clear reference values. Group sizes were also unspecified. Wang Y, et al. [23] used non-random allocation, placing polylactic acid-hydroxyapatite (PLA-HA) scaffolds with vascular endothelial growth factor (VEGF) in left calvarial defects and without VEGF in right defects, although random housing was implied. NBA results were missing, and discrepancies were noted between reported values and graphical data for VN and VA. Other studies omitted BMD [26], VN and BMD [24], and VN, BV/TV, and BMD [23]. Zheng et al. [27] reported Microfil-derived vascular volume/connectivity rather than VA or VN (and provided micro-CT BV/TV/BMD), and Yang et al. [29] reported Microfil VV/TV (not VA) and BMD on a relative (%) scale; VN was not provided. However, all reported at least one vascularisation and one bone formation outcome, mitigating the risk of selective reporting. Six studies [20,21,22,23,24,26] created two defects per rat. Four [20,21,22,24] implanted identical scaffolds in both defects, and one [26] used both scaffold types per rat, raising potential bias concerns. Only Wang G, et al. [26] employed random scaffold placement and was classified as low risk in this category. Zheng et al. [27] and Yang et al. [29] each created a single calvarial defect per rat. Accordingly, the comparisons presented in Table 2 should be interpreted as exploratory and hypothesis-generating rather than definitive estimates of effect.

## 4. Discussion

This systematic review synthesises factors influencing scaffold-driven vascularisation and bone regeneration in rodent calvarial defects evaluated by ‘Microfil’ perfusion and micro-CT.

### 4.1. Influence of Pore Size

Pore size and porosity strongly influence vascularisation, osteogenesis, and mechanics [19,30,31]. Larger pores (≥400 µm) were associated with higher BV/TV (*p* = 0.029), whereas VA showed only a positive, non-significant trend (*p* = 0.053) [32]. The included studies primarily report imaging-derived endpoints and do not directly measure transport properties or molecular signalling. Therefore, the mechanistic considerations below are hypothesis-generating and informed by the broader literature. One membrane study was retained for qualitative synthesis but excluded from statistical comparisons due to missing pore-size data [26]; a hierarchical scaffold study was also excluded from these analyses [21]. High porosity (60–90%), together with macropores (200–800 μm) and micropores (<10 μm), may better mimic bone; macropores support cell penetration whereas micropores facilitate ion exchange and protein adsorption [33]. Hierarchical β-TCP scaffolds with fibrous micropores reported increased vessel formation and higher BV/TV [34]. Mechanistically, it has been proposed that larger and better-interconnected pores may increase permeability and reduce transport tortuosity, which could support nutrient and oxygen delivery and facilitate endothelial migration and anastomosis. Conversely, smaller or poorly connected pores may delay vascular network maturation [32]. In addition, scaffold degradation may widen inter-pore throats and expose bioactive ions that could influence pro-angiogenic pathways (including VEGF- and HIF-related signalling), thereby potentially linking angiogenesis and osteogenesis [6,23,35,36]. VEGF loading may also promote angiogenesis, although excessive levels have been associated with dysfunctional vascular responses [37,38]. These mechanisms were not directly assessed in the included studies and should be interpreted as plausible explanations rather than evidence derived from this dataset. All in all, ≥400 µm, well-interconnected pores were associated with higher BV/TV and showed a positive VA trend. However, the VA difference did not reach statistical significance in this dataset. These architectures may improve mass transport and support osteogenesis, but a consistent vascular benefit cannot be concluded from the available VA data.

### 4.2. Role of Composite Scaffolds

Minimal new bone development was observed in blank composite scaffolds, particularly mesoporous bioactive glass (BG) mixed with poly(3-hydroxybutyrate-co-3-hydroxyhexanoate) and tricalcium phosphate-hydroxyapatite, and in control groups [21,23,25]. By contrast, a blank mesoporous BG-poly(lactide-co-glycolic acid) (PLGA) scaffold yielded significantly greater new bone formation and mineralisation than a simple non-composite PLGA scaffold and controls, with the highest levels observed when the composite was supplemented with the bioactive lipid FTY720 [24]. Several investigations indicate that composite scaffolds may outperform single-material designs [22,24,39]. However, in our endpoint-specific analysis, only NBA differed significantly between composite and non-composite scaffolds, whereas VN, VA, and BV/TV did not. In line with this, a core–shell collagen/mesoporous-silica composite delivering SR1 [29] produced higher BV/TV, relative BMD, and Microfil-derived VV/TV than blank or polymer-only controls, indicating that composite architecture coupled to targeted bioactivity augments both vascularisation and osteogenesis. Similarly, an IL-4-modulated matrix improved Microfil vascular indices, BV/TV, and BMD versus unmodified material, whereas excessive dosing (100 ng) attenuated these benefits [27], highlighting the importance of dose-tuned immunomodulation within composite systems. In this review, ‘non-composite (single-phase)’ denotes a scaffold composed of one material phase only, and ‘composite’ denotes constructs that integrate at least two distinct phases within the scaffold (for example, by blending, layering, core–shell architecture, or reinforcement); surface coatings were treated as functional modifications and did not reclassify a single-phase scaffold as composite. Further, synergy between bioactive and polymeric phases likely underpins these gains: bioactive glass-chitosan composites enhanced osteogenic differentiation and mineralisation relative to single-component controls, and collagen-hydroxyapatite constructs improved mechanical stability and osteoconductivity compared with constituent alone [40]. Material properties remain decisive: metals offer high strength and biocompatibility; BG and ceramics such as hydroxyapatite are strongly osteoconductive/osteoinductive but brittle as monoliths; natural polymers provide biocompatibility with limited mechanics; synthetic polymers allow property control yet are often hydrophobic and lack cell-recognition sites, so rational combinations are required to balance mechanics and bioactivity [1,36,41]. Overall, well-designed composites may provide advantages over single-material scaffolds. However, the present outcome-specific comparisons do not demonstrate consistent superiority across vascular and micro-CT endpoints. Specifically, composite status was not associated with significant differences in VN (*p* = 0.601), VA (*p* = 0.490), or BV/TV (*p* = 0.333), and BMD could not be robustly compared due to limited reporting (Table 2), so any vascular advantage attributable to composition alone should be interpreted cautiously. Nevertheless, the present dataset supports a statistically robust difference only for NBA, and further adequately powered, standardised comparisons are required. These findings support composite scaffolds and call for direct, parallel-arm comparative studies with standardised designs and outcomes to identify optimal material pairings and functional modifications for critical-size defect repair [39].

### 4.3. Effect of Scaffold Modifications (Dual Modifications)

Multiple studies evaluated angiogenic or osteogenic factors, cells, and small-molecule approaches, and generally reported directionally higher vascular and bone outcomes [20,21,23,24,25,26,28,42]. However, effects were heterogeneous across endpoints and not uniformly significant in the outcome-specific comparisons (Table 2). Single-factor modifications (e.g., VEGF, bone morphogenetic protein-2 (BMP-2), and fibroblast growth factor (FGF-2)) typically promote blood vessel growth and new bone production, but dual-factor loading (e.g., BMP-2 with VEGF or FGF-2) often outperforms single-factor delivery for VA, VN and bone regeneration outcomes [21,25,29,43]. In particular, dose-tuned IL-4-mediated M2 polarisation improved Microfil-derived vascular indices, BV/TV, and BMD in calvarial defects; however, excessive IL-4 dosing reduced these gains, highlighting the significance of controlled release and dosing windows. These complementary strategies that combine immunomodulation with architectural/compositional design also demonstrate benefits (Zheng et al., 2018) [27]. Moreover, scaffolds modified with magnesium silicate (MS) nanosheets have demonstrated enhanced vascularisation and osteogenesis when compared to blank composites [22,24,44,45]. For instance, Wu et al. [44] showed that adding calcium MS to silk fibroin and graphene oxide improved osteogenic differentiation and angiogenesis, whereas Yang J, et al. [29] found that micro-RNA-146a-loaded MS nanospheres impart dual osteogenic and immunoregulatory effects. Similarly, core–shell collagen/mesoporous-silica constructs that allowed for the prolonged release of the AhR inhibitor SR1 raised VV/TV (Microfil), BV/TV, and relative BMD, which is in line with the growth of CD34^+^ progenitors and the maturation of the vascular bed (e.g., α-SMA) [29]. Across multiple studies, dual-factor or dual-cell approaches (e.g., combining BMP-2 with VEGF, or delivering ASCs and EPCs together) appear superior to single modifications, with higher BV/TV and vessel metrics [21,25,28]. However, high-dose BMP-2 can cause adverse responses, underscoring the necessity of balanced, controlled-release delivery and synergistic co-loading strategies [46,47]. Although these combinations have shown promise, further large-scale, standardised investigations remain essential to determine the optimal dosages, delivery timelines, and release profiles for maximising angiogenic and osteogenic outcomes in critical-size defect repair.

### 4.4. Clinical Relevance and Future Directions

VN showed the strongest association with NBA, followed by BV/TV and BMD; VA was weaker/model-dependent, suggesting that increasing functional micro vessel number and bone volume may be more informative design targets than absolute vessel area [48,49]. These findings align with the literature emphasising the dual role of vascularisation—both as an essential component of bone healing and as a potential regulator of remodelling dynamics [50]. In terms of mechanism, dose-adjusted IL-4-mediated M2 polarisation and progenitor-driven vasculogenesis through SR1 enhanced VV/TV, BV/TV, and BMD in calvarial defects, while excessive cytokine dosing diminished these advantages, highlighting that “more vessels” is less critical than “more functional, mature vessels” [27,39]. Nevertheless, studies have also reported conflicting results regarding the threshold effects of vascularisation, suggesting that optimal angiogenesis levels rather than absolute increases in VA are critical for bone regeneration [35]. From a translational perspective, the present findings support scaffold architecture as a modifiable lever to influence perfusion and early vessel ingrowth within the rodent calvarial Microfil and micro-CT model. However, clinical relevance to implant dentistry and craniofacial reconstruction should be interpreted cautiously, because human defects differ in scale, biomechanics, healing compartments, and host factors that are not captured in this preclinical setting. Accordingly, these implications are presented as hypothesis-generating and require confirmation in larger-animal and clinically oriented studies. Therefore, we avoid direct clinical claims and emphasise that the conclusions primarily apply to imaging-defined outcomes in rodent critical-size calvarial defects. Future studies should consider integrating longitudinal assessments of vascular dynamics and mechanistic analyses of angiogenic–osteogenic coupling to better understand scaffold-driven bone regeneration and optimise vascularisation strategies for improved clinical outcomes.

### 4.5. Study Limitations and Risk of Bias

#### 4.5.1. Limitations

Variation in rat sex, age, strain, and defect size may have influenced healing responses. Prior evidence suggests greater new bone formation in 9 mm defects, likely due to younger adult rats being used. To minimise spontaneous repair in large defects, animals aged ≥ 16 weeks are recommended [51,52]. Most experiments used 8–13-week males, a choice that may have affected bone repair. Sex effects remain untested, and strain differences (e.g., Sprague–Dawley vs. Wistar) could also modulate outcomes, although current evidence is inconclusive [20,22,23,24,25,26,51,53]. Fewer than three studies per strain reported each primary outcome; therefore, we did not conduct formal Sprague–Dawley vs. Wistar subgroup analysis or meta-regression, and sex effects were not amenable to quantitative comparison. Age-based subgrouping was infeasible due to insufficient per-stratum counts.

The timing of ‘Microfil’ perfusion and micro-CT after implantation likely influenced our results. For example, Kuttappan et al. [28] and Tu et al. [25] reported greater new bone formation with longer implantation periods; some studies also noted higher VN/VA at later endpoints. However, early trends sometimes anticipated later outcomes, and differences were not universal. In addition, selective and incomplete reporting of core outcomes (VN, VA, BV/TV, BMD, and NBA) reduced the effective sample size for each comparison and limits the robustness and generalisability of the quantitative findings. Endpoint heterogeneity limited analysis: multiple studies documented Microfil-derived vascular volume or connectivity instead of VA/VN, or used relative BMD units, preventing direct grouping [27,29]. Non-standardised perfusion and micro-CT parameters (perfusion pressure and volume, Microfil curing, voxel size, and segmentation thresholds) likely introduced measurement variation [27,29]. The inconsistent reporting of pore size and porosity in composite or core–shell designs restricted architecture-based subgrouping [29]. Ultimately, the immunomodulatory effects were dependent on both dosage and time; for instance, IL-4 exhibited a non-linear response characterised by reduction at higher levels [27].

Moreover, restricting inclusion to rodent calvarial critical-size defects limits generalisability to other sites and species. Calvarial defects are largely non-load-bearing and differ from long-bone defects in biomechanics, healing compartments, and fixation requirements, which can independently influence vascular and bone outcomes [15,16]. Requiring Microfil perfusion with micro-CT also excludes studies using alternative vascular assessment methods. Therefore, conclusions apply primarily within this imaging-defined calvarial model scope.

The evidence base was limited, with only 10 studies meeting the established inclusion and exclusion criteria. This constrained the statistical power for subgroup analyses and meta-regression, diminished the precision of pooled estimates, and increased the susceptibility to small-study effects and outcome reporting bias, especially due to the selective reporting of vascular and bone endpoints. Future work could strengthen and broaden the evidence base by (i) widening eligibility criteria to include studies that quantify vascularisation using validated alternatives to Microfil-based micro-CT (e.g., immunohistochemical endothelial markers, other contrast agents, or complementary imaging modalities), (ii) expanding scaffold scope to incorporate additional 3D-printed architectures and material classes beyond the composite or core–shell designs captured here, and (iii) extending time frames and search coverage to capture earlier foundational studies and emerging designs, as well as longer follow-up periods to improve comparability of late-stage outcomes. In parallel, adoption of more standardised reporting for perfusion parameters, micro-CT acquisition and segmentation, and a minimum core outcome set (including raw units and variance measures) would materially improve future quantitative synthesis.

#### 4.5.2. Risk of Bias

Risk of bias was low or unclear for most domains, but three studies had ≥1 high-risk items, reporting gaps—such as missing VN/BV/TV/BMD values or unreferenced fold-change VA—and absent details on allocation concealment, random housing, and blinding reduced evaluability [20,26,28]. These issues underscore the need for transparent methods, standardised outcomes and time points, inclusion of females to assess sex effects, and adequate power to confirm associations between scaffold characteristics and vascularisation or bone outcomes. In addition, Wang Y. et al. (2023) showed discrepancies between figure-based and text-reported VN/VA values, and we therefore treated these outcomes as figure-digitised estimates, which increases uncertainty for that study-level contribution [23].

Overall, optimised pore architecture, composite formulations, and rational dual modifications appear most promising for enhancing neovascularisation and bone regeneration, warranting confirmation in adequately powered, standardised preclinical studies.

## 5. Conclusions

Composite 3D-printed scaffolds, particularly those with larger pore sizes (≥400 µm) and rational dual modifications, are associated with improved bone regeneration in rodent calvarial defect models; evidence for a vascular benefit of larger pores was limited to a non-significant positive VA trend. However, the certainty of this effect is constrained by small study sizes, heterogeneity of scaffold designs and animal models, and incomplete reporting. Therefore, quantitative comparisons should be considered exploratory. To strengthen the evidence base, future work should adopt standardised protocols for Microfil perfusion and micro-CT with explicitly reported acquisition and analysis parameters and harmonised time points; employ a core outcome set: VN, VA, BV/TV, BMD, and NBA, using uniform operational definitions; incorporate vascular network quality and maturity readouts (e.g., VV/TV, connectivity, radius distributions); be adequately powered, preregistered, and include both sexes and relevant strains; and undertake head-to-head comparisons of material and architecture combinations and functional modifications. In summary, 3D-printed composite scaffolds with larger pore sizes (≥400 µm) and well-tuned angiogenic, osteogenic, and or immuno- and progenitor-modulating factors appear to enhance bone and blood vessel regeneration in critical-size defects, but larger, standardised studies are needed to confirm these results.

## Figures and Tables

**Table 1:** Key data extracted from included studies: animal models, groups, defect size, time points, measured outcomes.

**Yang et al., 2024 [29]**	Female Sprague–Dawley rats, mature	Not given	3 (CT; NP@Sc; SNP@Sc)	Ø 5 mm (1 defect/rat)	In vivo: Microfil, Micro-CT, histology; vascular IHC	4 weeks	2 and 4 weeks	4 weeks	Microfil-μCT angiography: VV/TV% at 4 w—CT 15.94 ± 4.62%, NP@Sc 19.05 ± 2.64%, SNP@Sc 32.67 ± 6.51%. Vascular IHC: α-SMA+ area (%) 2 w—CT 0.33 ± 0.27, NP@Sc 0.07 ± 0.03, SNP@Sc 0.83 ± 0.26; 4 w—CT 0.53 ± 0.08, NP@Sc 0.10 ± 0.06, SNP@Sc 1.08 ± 0.12. (CD34/EPC marker panel increased in SNP group.)	BV/TV (%): 2 w—CT ≈ 13.01; NP@Sc ≈ 11.30; SNP@Sc ≈ 15.75. 4 w—CT ≈ 17.00; NP@Sc ≈ 15.54; SNP@Sc ≈ 23.91. BMD: relative (%) units (not directly comparable)	Nanoparticle modification (NP@Sc; SNP@Sc)	Ø 5 mm; other design metrics not given	Not given
**Zheng et al., 2018 [27]**	tMale SD rats, mature	Not given	4 (Control [0 ng IL-4]; IL-4 low; IL-4 medium; IL-4 high)	Ø 5 mm (1 defect/rat)	In vivo: Microfil vascular casting, Micro-CT, histology; IHC	12 weeks	6 and 12 weeks	12 weeks	Microfil-μCT angiography (12 w): vessel volume fraction (vascular volume), microvessel connectivity, and vessel thickness quantified; VN/VA not reported. 10 ng IL-4 showed the highest vessel volume, connectivity, and thickness	NBA (%): ≈ 10.9 (0 ng), 31.7 (10 ng), 22.3 (50 ng), 7.2 (100 ng). BV/TV: 10 ng highest (1.23–5.05× vs. other groups). BMD: 10 ng highest (1.33–3.79× vs. other groups)	IL-4 (dose-dependent loading)	Ø 5 mm; other design metrics not given	Not given
**Qi X. et al. 2017 [21]**	Male SD rats, mature	24 animals (n = 6 per group)	4 (Group A: PHMG; Group B: PHMB; Group C: PHMD; Group D: PHMBD)	Ø 5 mm (2 defects per rat)	In vivo: Microfil, Micro-CT, histology, fluorescence labelling, and immunohistochemical analysis; in vitro: RT-qPCR analysis, Western blotting	8 weeks	8 weeks	2, 4, and 6 weeks	VN: PHMG ≈ 3; PHMB ≈ 30; PHMD ≈ 40; PHMBD ≈ 90; VA in %: PHMBD ≈ 86.09; PHMD ≈ 36.11; PHMB ≈ 21.65; PHMG ≈ 1.27	NBA (in %): PHMBD ≈ 89.5; PHMG ≈ 4.50; PHMB ≈ 26.17; PHMD ≈ 14.00; BV/TV (in %): PHMG ≈ 3; PHMB ≈ 35; PHMD ≈ 15; PHMBD ≈ 53; BMD (in g/cm^3^): PHMG < 0.1; PHMB ≈ 0.5; PHMD ≈ 0.3; PHMBD 0.88	DMOG, rhBMP-2	5 × 5 mm; macropores 450–900 μm, micropores 20–50 μm (on frame walls); porosity 80%	MBG-PHBHHx (PHMG)
**Min Z. et al. 2015 [20]**	Male SD rats, 12-week-old	12 animals (n = 6 for G1, G2)	3 (Group A: PHMG; Group B: PHMD; Group C: control)	Ø 5 mm (2 defects per rat)	In vivo: Microfil, Micro-CT, histology, sequential fluorescence labelling; in vitro: cell attachment, cytotoxicity, ALP activity, RT-qPCR analysis	8 weeks	8 weeks	2, 4, and 6 weeks	not specified	not specified	DMOG	6 × 3 mm; cylindrical shape; well-defined square pore structure (ordered, uniform mesoporous), 700 μm between strands	MBG-PHBHHx (PHMG)
**Kuttappan S. et al. 2018 [28]**	Male Wistar rats, 4–5-month-old	Not given	6 (Group A: Sc; Group B: Sc/B; Group C: Sc/V; Group D: Sc/F; Group E: Sc/B/V; Group F: Sc/B/F)	Ø 8 mm (1 defect per rat; thickness 1.5 mm)	In vivo: Microfil, Micro-CT, histology, and histomorphometry; in vitro/in vivo: growth factor release, cytocompatibility, osteogenic differentiation, endothelial functionality	4 weeks	4 weeks	4 and 12 weeks	VN: not specified; VA in fold increase (in regard to control): Sc < 1; ScB ≈ 2; ScV > 3 < 4; ScF ≈ 2; ScBV ≈ 4; ScBF > 4 < 5	NBA (in %): Control ≈ 0 at 4 w., 1,5 at 12 w.; Sc ≈ 3 at 4 w., 37 at 12 w.; ScB ≈ 48 at 4 w., 80 at 12 w.; ScV ≈ 4 at 4 w., 45 at 12 w.; ScF ≈ 3 at 4 w., 51 at 12 w.; ScBV ≈ 52 at 4 w., 87 at 12 w.; ScBF ≈ 56 at 4 w., 92 at 12 w.; BV/TV and BMD not specified	BMP2, FGF2, VEGF	8 × 1.5 mm; pore size 50–350 µm; porosity 58.8% ± 7.3%; overall compressive strength of 28 ± 3.5 MPa	nanoHA-silica gelatinous matirx-PLLA (Sc)
**Sun T.W. et al. 2017 [22]**	Male SD rats, 8-week-old	24 animals (n = 8 per group)	3 (Group A: CS; Group B: HANWs/CS; Group C: HANW@MS/CS)	Ø 5 mm (2 defects per rat)	In vivo: Microfil, MicroCT, histology; in vitro: SEM, TEM, and XRD, drug loading analysis, analysis of cytoskeleton staining and SEM micrographs, RT-qPCR analysis	12 weeks	12 weeks	12 weeks	VN: CS ≈ 13; HANWs/CS ≈ 29; HANW@MS/CS ≈ 50; VA in %: CS 5.38%; HANWs/CS ≈ 9.66%; HANW@MS/CS ≈ 13.26%	NBA (in %): CS ≈ 3.15; HANWs/CS ≈ 22.99; HANW@MS/CS ≈ 39.41; BV/TV (in %): CS ≈ 4.92; HANWs/CS ≈ 25.06; HANW@MS/CS ≈ 40.15; BMD not specified	Not given	5 × 2 mm; pore sizes 200–300 μm; compressive strength: HANW@MS/CS 6.18, HANWs/CS 7.84, pure CS 5.38 kPa	CS/CS-HANWs/CS-HANW@MS
**Wang Y. et al. 2023 [23]**	Male SD rats, 12-week-old	12 animals (n = 6 at 4 and 8 weeks)	2 (Group A: PLA/HA; Group B: VEGF + PLA/HA)	Ø 5 mm (2 defects per rat)	In vivo: Microfil, Micro-CT, histology; in vitro: immunofluorescence staining, scanning electron microscopy, energy spectrum analysis	4 and 8 weeks	4 and 8 weeks	4 and 8 weeks	VN: PLA/HA ≈ 16 at 4 w., 17 at 8 w.; PLA/HA + VEGF≈ 24 at 4 w., 25 at 8 w; VA in %: PLA/HA ≈ 25 at 4 w., 35 at 8 w.; PLA/HA + VEGF ≈ 38 at 4 w., 64 at 8 w. (Values digitised from figures due to inconsistency between figure and text reporting in the original.)	NBA not specified; BV/TV (in %): PLA/HA ≈ 0.03 at 4 w., 0.1 at 8 w.; PLA/HA + VEGF ≈ 0.03 at 4 w., 0.13 at 8 w.; BMD (in g/cm^3^): PLA/HA ≈ 1.03 at 4 w., 1.05 at 8 w.; PLA/HA + VEGF ≈ 1.05 at 4 w., 1.10 at 8 w.	VEGF	5 mm; circular membrane, irregular spun woven structures, uniform spinning morphology; homogenous pores; porosity 63%	PLA-HA
**Li S. et al. 2019 [24]**	Male SD rats, 8-week-old	24 animals (n = 6 (3 for Microfil) per group)	4 (Group A: control; Group B: PLGA; Group C: MBG-PLGA; Group D: FTY/MBG-PLGA)	Ø 5 mm (2 defects per rat)	In vivo: Microfil, Micro-CT, histology, sequential fluorescence labelling, immunofluorescence assay of CD31 and Emcn; in vitro: angiogenesis assay of HUVECs	8 weeks	8 weeks	4 and 6 weeks	VN: not specified; VA in %: Control ≈ 1.5; PLGA ≈ 4.10; MBG-PLGA ≈ 10.25; FTY/MBG-PLGA ≈ 21.07	NBA (in %): Control ≈ 1; PLGA ≈ 2.83; MBG-PLGA ≈ 7.81; FTY/MBG-PLGA ≈ 16.6; BV/TV (in %): Control ≈ 2.5; PLGA ≈ 4; MBG-PLGA ≈ 9.15; FTY/MBG-PLGA ≈ 17.47; BMD not specified	FTY	12 × 2 mm; pore size 257 ± 50 μm (PLGA), 252 ± 45 μm (MBG-PLGA); porosity 81% (PLGA), 82% (MBG-PLGA)	PLGA/ PLGA-MBG
**Wang G. et al. 2017 [26]**	Male SD rats, 12-week-old	16 animals (n = 4 per group)	4 (Group A: TCP/HA; Group B: Sr-HT-Gahnite; Group C: TCP/HA + ASCs; Group D: Sr-HT-Gahnite + ASCs)	Ø 5 mm (2 defects per rat)	In vivo: Microfil, fluorochrome labelling histomorphometric analysis, histology; in vitro: ASCs culture, semi-quantitative study (ALP activity), Alizarin Red S staining, Ion concentrations (ICP-AES), expression of angiogenic genes, HUVECs culture (MTT assay, transwell assay, mRNA expression levels)	8 weeks	8 weeks	4, 6, and 8 weeks	VN: not specified; VA in %: TCP/HA ≈ 1.9; TCP/HA/ASCs ≈ 2.8; Sr-HT-gahnite ≈ 3.0; Sr-HT-gahnite/ASCs ≈ 6.7	NBA (in %): TCP/HA ≈ 0.5; TCP/HA/ASCs ≈ 6.6; Sr-HT-gahnite ≈ 5.8; Sr-HT-gahnite/ASCs ≈ 14.5; BV/TV and BMD not specified	ASCs	Defect 5 mm; pore size 400–700 μm (TCP/HA), 500 μm (Sr-HT-gahnite); porosity 91% (TCP/HA), 85% (Sr–HT–gahnite)	TCP-HA/ Sr-HT-Gahnite
**Tu C. et al. 2020 [25]**	Male SD rats, 12–13-week-old	126 animals (n = 24 (6 for Microfil) per group)	5 (Group A: control; Group B: PLA-HA; Group C: PLA-HA/EMF; Group D: PLA-HA/BMSCs; Group E: PLA-HA/BMSCs/EMF)	Ø 6 mm (1 defect per rat)	In vivo: Microfil, Micro-CT, histology, biomechanical analysis; in vitro: scanning electron microscopy, CCK-8 assay and a LIVE/DEAD kit	6 weeks	4 and 12 weeks	4 and 12 weeks	VN: Control ≈ 5; Scaffold ≈ 20; Scaffold/EMF ≈ 35; Scaffold/BMSCs ≈ 40; Scaffold/BMSCs/EMF ≈ 80; VA in %: Control ≈ 2.5; Scaffold ≈ 10; Scaffold/EMF ≈ 21; Scaffold/BMSCs ≈ 23; Scaffold/BMSCs/EMF ≈ 35	NBA (in %): Control ≈ 3 at 4 w., 5 at 12 w.; Scaffold ≈ 10 at 4 w., 21 at 12 w.; Scaffold/EMF ≈ 20 at 4 w., 40 at 12 w.; Scaffold/BMSCs ≈ 21 at 4 w., 41 at 12 w.; Scaffold/BMSCs/EMF ≈ 30 at 4 w., 58 at 12 w.; BV/TV (in %): Control ≈ 2 at 4 w., 5 at 12 w.; Scaffold ≈ 9 at 4 w., 21 at 12 w.; Scaffold/EMF ≈ 20 at 4 w., 43 at 12 w.; Scaffold/BMSCs ≈ 23 at 4 w., 48 at 12 w.; Scaffold/BMSCs/EMF ≈ 34 at 4 w., 73 at 12 w.; BMD (in mg/cm^3^): Control ≈ 20 at 4 w., 50 at 12 w.; Scaffold ≈ 70 at 4w., 170 at 12 w.; Scaffold/EMF ≈ 160 at 4w, 320 at 12 w.; Scaffold/BMSCs ≈ 180 at 4w., 330 at 12 w.; Scaffold/BMSCs/EMF ≈ 260 at 4w., 430 at 12w.	EMF, BMSCs	6 × 0.6 mm; cylindrical shape; pore diameter 1000 μm; porosity 70 ± 2.23%; compression strength 31.18 ± 4.86 MPa	PLA-HA
**Author(s) and year**	**Animals**	**Sample Size**	**Study Groups**	**Defect Features**	**Analysis Methods**	**Timing of Microfil**	**Timing Micro-CT**	**Timing Histology**	**Vascularisation Analysis: VN, VA**	**New Bone Formation Analysis: NBA, BV/TV, BMD**	**Scaffold Modification**	**Scaffolds Design: measures, geometry, pore size, porosity, compressive strength**	**Scaffold Composition**

**Table 2:** Statistical comparison of pore size, composite status, presence and number of modifications for measured outcomes; analyses are outcome-specific and include only studies reporting directly comparable endpoint definitions and units (n varies by outcome).

Factor	Independent-Samples *t*-Test for Equality of Means					Cohen’s d		
	Variable	t-Value	*p*-Value	95% CI Lower *	95% CI Upper *	Effect Size **	95% CI Lower	95% CI Upper
Pore Size (≥400 µm/<400 µm)	VN	−0.74	0.491	−58.35	32.18	23.05	−2.08	0.99
Composite (Yes/No)		0.57	0.601	−42.03	63.63	17.37	−1.6	2.77
Mod (Yes/No)		2.19	0.051	−0.15	53.3	21.83	−0.01	2.24
Number of Mod (Single/Dual)		−9.19	<0.001	−65.27	−36.73	6.63	−12.56	−2.8
Pore Size (≥400 µm/<400 µm)	VA	−2.28	0.053	−23.42	0.16	7.92	−2.88	0.02
Composite (Yes/No)		0.72	0.49	−12.71	24.32	10.16	−1.02	2.13
Mod (Yes/No)		2.58	0.025	3.39	41.33	19.4	0.19	2.09
Number of Mod (Single/Dual)		−2.08	0.071	−75.85	3.84	21.86	−3.35	0.13
Pore Size (≥400 µm/<400 µm)	BV/TV	−2.67	0.029	−54.94	−3.98	17.12	−3.2	−0.17
Composite (Yes/No)		1.05	0.333	−15.81	39.71	13.9	−0.84	2.5
Mod (Yes/No)		2.03	0.055	−0.39	31.43	0.87	−0.01	1.75
Number of Mod (Single/Dual)		−2.51	0.046	−72.27	−0.87	17.87	−3.95	−0.03
Pore Size (≥400 µm/<400 µm)	NBA	0.02	0.981	−35.8	36.6	29.23	−1.12	1.15
Composite (Yes/No)		2.7	0.031	1.78	26.99	14.1	−0.63	2.62
Mod (Yes/No)		2.23	0.038	1.19	36.29	0.95	0.06	1.84
Number of Mod (Single/Dual)		−3.89	0.002	−75.13	−21.15	20.94	−3.74	−0.8
Mod (Yes/No)	BMD	0.42	0.682	−0.49	0.72	0.38	−1.08	1.65
Number of Mod (Single/Dual)		−0.54	0.627	−0.87	0.58	0.34	−2.07	1.25

* 95% confidence interval of the mean difference. ** Cohen’s d calculated using the pooled standard deviation. Note: For VN, BV/TV, BMD and NBA equal variances were assumed (Levene *p* > 0.05). For VA Levene’s test indicated unequal variances (*p* = 0.038); therefore, *t*-tests with unequal variances (Welch correction) were used. For transparency, analyses were restricted to studies with extractable quantitative data for the specific endpoint; consequently, some outcomes and comparisons include fewer studies due to non-reporting or non-comparable reporting formats (for example, fold-change VA or alternative Microfil metrics). For transparency, analyses were restricted to studies with extractable quantitative data using directly comparable endpoint definitions and units; therefore, outcomes reported as fold-change VA, VV/TV or connectivity, or relative or non-calibrated BMD were not pooled and were summarised descriptively.

## Data Availability

No new data were created or analyzed in this study. Data sharing is not applicable to this article.

## Abbreviations

The following abbreviations are used in this manuscript:

ALP	Alkaline Phosphatase
BG	Bioactive Glass
BMD	Bone Mineral Density
BMP	Bone Morphogenetic Protein
BMSC	Bone Marrow-Derived Mesenchymal Stem Cell
BV/TV	Bone Volume Fraction
CS	Chitosan
CT	Computed Tomography
DMOG	Dimethyloxalylglycine
EMF	Electromagnetic Fields
EPC	Endothelial Progenitor Cells
FGF	Fibroblast Growth Factor
FTY	Bioactive Lipid FTY720
GF	Growth Factor
HA	Hydroxyapatite
HT	Hardystonite
HUVEC	Human Umbilical Vein Endothelial Cell
M	Mean Value
MS	Magnesium Silicate
MTT	3-(4,5-dimethylthiazol-2-yl)-2,5-diphenyltetrazolium bromide
NBA	New Bone Area
NW	Nanowire
PHBHHx	Poly(3-hydroxybutyrate-co-3-hydroxyhexanoate)
PHMG	Combination of mesoporous BG and PHBHHx
PLA	Polylactic Acid
PLGA	Poly(lactide-co-glycolic acid)
PLLA	Poly-L-lactic acid
RT-qPCR	Real-Time Quantitative Polymerase Chain Reaction
SD	Sprague–Dawley
SEM	Scanning Electron Microscopy
Sr	Strontium
StD	Standard Deviation
TCP	Tricalcium Phosphate
TEM	Transmission Electron Microscopy
VA	Vascularised Area
VEGF	Vascular Endothelial Growth Factor
VN	Vessel Number
XRD	X-ray Diffraction
3D	Three-Dimensional
